# Prevalence and risk factors of chronic kidney disease and diabetic kidney disease in Chinese rural residents: a cross-sectional survey

**DOI:** 10.1038/s41598-019-46857-7

**Published:** 2019-07-18

**Authors:** Jiayu Duan, Chongjian Wang, Dongwei Liu, Yingjin Qiao, Shaokang Pan, Dengke Jiang, Zihao Zhao, Lulu Liang, Fei Tian, Pei Yu, Yu Zhang, Huanhuan Zhao, Zhangsuo Liu

**Affiliations:** 1 Department of Nephrology, The First Affiliated Hospital of Zhengzhou University, Research Institute of Nephrology, Zhengzhou University, Zhengzhou, 450052 China; 2 Department of Epidemiology and Biostatistics, College of Public Health, Zhengzhou University, Zhengzhou, Henan P.R. China

**Keywords:** Chronic kidney disease, Lifestyle modification

## Abstract

We conducted a cross-sectional survey including 23869 participants and aimed to measure the prevalences of and risk factors for chronic kidney disease (CKD) and diabetic kidney disease (DKD) in a Chinese rural population. CKD and DKD status was defined according to the combination of estimated glomerular filtration rate (eGFR) and presence of albuminuria Participant completed a questionnaire involving life-style and relevant medical history, and the blood and urinary specimen were taken. The age- and gender- adjusted prevalences of CKD and DKD were calculated and risk factors associated with the presence of CKD and DKD were analyzed by logistic regression. The overall prevalence of CKD was 16.4% (15.9–16.8%) and of DKD was 2.9% (2.7–3.1%). In participants with diabetes, the overall prevalence of CKD was 35.5% (95% CI = 33.7–37.3%). Factors independently associated with renal damage were age, gender, education, personal income, alcohol consumption, overweight, obesity, diabetes, hypertension and dyslipidemia. Our study shows current prevalences of CKD and DKD in Chinese rural residents. Further researches could identify potential factors explaining the observed differences and implement the interventions to relieve the high burden of CKD and DKD in rural population.

## Introduction

Chronic kidney disease (CKD) has been a global public health issue in the past decades and affects more than 10% population worldwide. People with diabetes and hypertension are exposed to 50% risk of developing CKD^1^. Unhealthy diet, unbefitting physical activity and obesity are also reported to be associated with the increasing risk of CKD^2^. The burden of CKD is not only restricted to the requirement of renal replacement therapy for end-stage renal disease (ESRD), but also its other serious outcomes, such as cardiovascular events and mortality, are strongly influenced by kidney involvement^3,4^. In 2010, the mortality caused by CKD almost doubled comparing with which in 1990 and it was ranked as the 18th risk factor in the mortality list^5^.

In China, CKD is highly prevalent since the rapid increasing prevalences of relevant risk factors, like diabetes, hypertension, unhealthy diet, unbefitting physical activity and metabolic syndrome^6–9^. The inadequate awareness and control of diabetes and hypertension exacerbate the socioeconomic and health burden of CKD in Chinese population^10–13^. In 2010, the prevalences of total of diabetes and prediabetes were 9.7% and 15.5%, respectively, which accounted for about 92.4 million adults with diabetes and 148.2 million adults with prediabetes^14^. Diabetes has replaced glomerulonephritis as the major cause of CKD in hospitalized population since 2011 and contributed to 1.23% diabetes-related CKD in general population^15^. Diabetic kidney disease accounted for 43.2% and 46.2% of ESRD patients in Taiwan and Hong Kong, respectively^16,17^. In 2012, the overall prevalence of CKD was 10.8%, estimating about 119.5 million Chinese adults with CKD^18^.

Nevertheless, the prevalence of hypertension has varied from 29.6% to 19.5% in the past five years^12^. Liu *et al*. reported that prevalence of diabetes was 6.6% in 2016, accounting for almost 90 million Chinese with diabetes^8^. Previous studies also have demonstrated that the prevalence of CKD varies substantially among geographical regions and time frames^19–22^. This diversity might be associated with the variability in diet pattern, lifestyle, education and economic condition. Therefore, we conducted a cross-sectional study to provide current data on the prevalences of chronic kidney disease, diabetic kidney disease and associated risk factors in the adult population in China.

## Results

A total of 23869 people were involved in this study. Generally, participants with lower eGFR, albuminuria and DKD performed older, lower educated and personal income, inadequate physical activities and consumption of fruits and vegetables, and higher prevalences of hypertension, dyslipidemia, diabetes hyperuricemia, than did participants without renal damage (Table 1).

**Table 1:** General characteristics of participants according to indicators of renal damage

	Participants with eGFR < 60 mL/min/1.73 m^2^ (N = 635)	Participants with albuminuria (N = 3958)	Participants with DKD (N = 962)	Participants without renal damage (N = 19522)	Total (N = 23869)
Age	67.4 (14.6)	58.5 (13.3)	70.0 (11.1)	55.7 (12.9)	56.4 (13.1)
Men	262 (41.3%)	1505 (38.0%)	369 (38.4%)	7927 (40.6%)	9597 (40.2%)
Education					
≤Primary school	457 (72.0%)	2257 (57.0%)	598 (62.2%)	9230 (47.3%)	11774 (49.3%)
Junior high school	77 (12.0%)	1105 (27.9%)	250 (26.0%)	6856 (35.1%)	7999 (33.5%)
≥Senior high school	101 (16.0%)	596 (15.1%)	113 (11.8%)	3433 (17.6%)	4096 (17.2%)
Per capita monthly income (RMB)					
≤500	193 (30.4%)	1188 (30.0%)	379 (39.4%)	5598 (28.7%)	6911 (29.0%)
500-	61 (9.6%)	933 (23.6%)	229 (23.8%)	5237 (26.8%)	6202 (26.0%)
≥1000	182 (28.7%)	1272 (32.1%)	284 (29.6%)	6335 (32.5%)	7749 (32.5%)
Current smoker	75 (11.8%)	594 (15%)	127 (13.2%)	3507 (18.0%)	4146 (17.4%)
Habitual drinker	54 (8.5%)	523 (13.2%)	115 (12.0%)	3194 (16.4%)	3752 (15.7%)
Dietary pattern					
Diet rich in fruits and vegetables	226 (35.6%)	1648 (41.6%)	368 (38.3%)	8471 (43.4%)	10258 (43.0%)
High fat diet	45 (7.1%)	575 (14.5%)	131 (13.6%)	3681 (18.9%)	4279 (17.9%)
Physical activity					
Low	421 (66.3%)	1732 (43.8%)	466 (48.5%)	7135 (36.6%)	9132 (38.3%)
Moderate	84 (13.2%)	1289 (32.6%)	289 (30.1%)	7336 (37.6%)	8668 (36.3%)
High	130 (20.5%)	937 (23.7%)	206 (21.4%)	5048 (25.9%)	6069 (25.4%)
Self-reported HBV infection	0 (0.0%)	33 (0.8%)	5 (0.5%)	168 (0.9%)	201 (0.8%)
Hypertension	283 (44.6%)	1865 (47.1%)	520 (54.1%)	4887 (25.0%)	6899 (28.9%)
Dyslipidemia	102 (16.1%)	1354 (34.2%)	456 (47.5%)	5227 (26.8%)	6628 (27.8%)
Hyperuricemia	177 (27.9%)	458 (11.6%)	103 (10.7%)	1262 (6.5%)	1814 (7.6%)
Diabetes	126 (19.8%)	908 (23.0%)	NA	1748 (9.0%)	2710 (11.4%)
Body mass index (kg/m^2^)	23.9 (3.7)	25.0 (3.9)	25.7 (3.8)	24.3 (3.4)	24.4 (3.5)
Total cholesterol (mmol/L)	5.2 (1.2)	4.9 (1.1)	5.1 (1.2)	4.5 (1.0)	4.6 (1.0)
Triglyceride (mmol/L)	1.7 (1.4)	1.9 (1.5)	2.4 (1.8)	1.6 (1.1)	1.6 (1.2)
LDL cholesterol (mmol/L)	3.2 (1.0)	3.0 (0.9)	3.0 (1.0)	2.9 (0.8)	2.9 (0.8)
HDL cholesterol (mmol/L)	1.4 (0.4)	1.3 (0.4)	1.3 (0.3)	1.4 (0.4)	1.4 (0.4)
FPG (mmol/L)	5.4 (2.2)	6.0 (2.4)	9.1 (3.1)	5.3 (1.4)	5.4 (1.7)
Uric acid (µmol/L)	334.6 (122.5)	287.1 (95.7)	282.6 (117.1)	272.2 (78.9)	275.5 (83.1)
Creatinine (µmol/L)	149.0 (110.7)	69.4 (37.8)	74.5 (54.1)	62.7 (13.9)	65.2 (26.7)
eGFR (mL/min/1.73 m^2^)	45.5 (14.4)	92.7 (19.4)	89.2 (23.3)	99.0 (13.6)	97.1 (16.3)
ACR (mg/g)	22.4 (13.1-59)	52.6 (37.7-92.7)	60.2 (38.8-116.9)	9.3 (4.5-15.3)	11.5 (5.5-21.8)

Note: Data were n (%) or mean (standard deviation) except for ACR, which was presented as median with interquartile range.

Abbreviations: DKD, diabetic kidney disease; HBV, hepatitis B virus; LDL, low density lipoprotein; HDL, high density lipoprotein; eGFR, estimated glomerular filtration rate; FPG, fast plasma glucose ACR, albumin: creatinine ratio; NA, not applicable.

**Figure 1:**
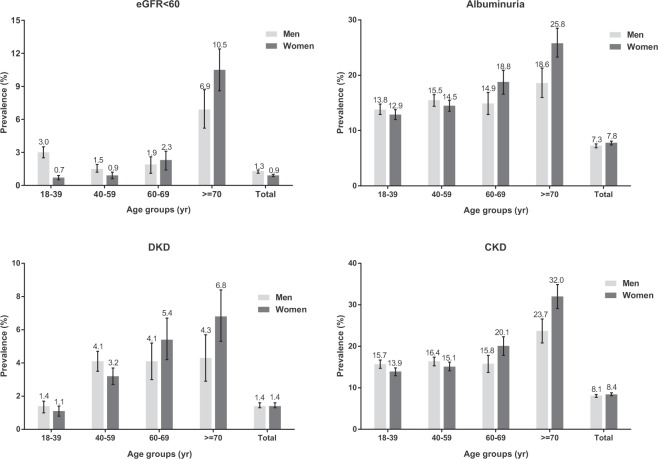
Adjusted prevalence of indicators of renal damage, diabetic kidney disease and chronic kidney disease, stratified by sex and age. Chronic kidney disease was defined as eGFR less than 60 mL/min/1.73 m^2^ or presence of albuminuria. Diabetic kidney disease defined as participants with diabetes and one or two indicators of renal damage. Bars were 95% confidence interval. CKD = chronic kidney disease, DKD = diabetic kidney disease, eGFR = estimated glomerular filtration rate.

According to the stratification of renal indicators, there were 635 individuals performed eGFR less than 60 mL/min/1.73 m^2^ and 3947 individuals performed albuminuria. Totally, 4347 participants were suffering from CKD, in which 962 subjects were DKD patients. The adjusted prevalence of eGFR less than 60 mL/min/1.73 m^2^ was 2.7% (95% CI = 2.5–2.9%) and that of albuminuria was 15.0% (95% CI = 14.6–15.5%). The overall prevalence of CKD and DKD was 16.4% (95% CI = 15.9–16.8%) and 2.9% (95% CI = 2.7–3.1%), respectively (Table 2). As shown in Fig. 1, comparing with men, prevalences of reduced eGFR and albuminuria were much lower in women who aged 18–39 (3.0 versus 0.7% and 13.8 versus 12.9%) and 40–59 (1.5 versus 0.9% and 15.5 versus 14.5%) years, while that was reversed in women subjects aged 60–69 (1.9 versus 2.3%) and ≥70 (6.9 versus 10.5%) years. Overall, the prevalences of CKD and DKD were increased with age in both men and women participants.

**Table 2:** Adjusted prevalence of indicators of renal function and chronic kidney disease, by disease stage.

Renal indicator								Chronic kidney disease (in 23869 participants)	
Stage	eGFR (mL/min/1.73 m^2^)			Albuminuria		Diabetic kidney disease			
		n	Prevalence (95% CI)	n	Prevalence (95% CI)	n	Prevalence (95% CI)	N	Prevalence (95% CI)
1	>90	17748	80.9 (80.4–81.4)	2561	14.4 (13.9–14.9)	592	3.3 (3.1–3.6)	2561	11.0 (10.6–11.4)
2	60–89	5486	17.0 (16.5–17.5)	1151	21.0 (19.9–22.1)	244	4.4 (3.9–5.0)	1151	3.1 (2.9–3.3)
3	30–59	530	1.3 (1.1–1.4)	195	36.8 (32.7–40.9)	94	17.7 (14.5–21.0)	530	1.3 (1.1–1.4)
3a	45–59	424	1.0 (0.9–1.1)	146	34.4 (29.9–39.0)	67	15.8 (12.3–19.3)	424	1.0 (0.9–1.1)
3b	30–44	106	0.3 (0.2–0.3)	49	46.2 (36.6–55.9)	27	25.5 (17.0–33.9)	106	0.3 (0.2–0.4)
4	15–29	57	0.4 (0.3–0.5)	32	56.1 (42.9–69.4)	21	36.8 (23.9–49.8)	57	0.4 (0.3–0.5)
5	<15	48	0.4 (0.4–0.5)	8	16.7 (5.7–27.6)	11	22.9 (10.6–35.2)	48	0.4 (0.3–0.5)
Total		23869	100	3947	15.0 (14.6–15.5)	962	2.9 (2.7–3.1)	4347	16.4 (15.9–16.8)

Note: Albuminuria was defined as urinary albumin to creatinine ratio > 30 mg/g creatinine. CKD was defined as an eGFR less than 60 mL/min/1.73 m^2^ or presence of albuminuria. All prevalences were adjusted for synthesized weights.

Abbreviations: eGFR, estimated glomerular filtration rate.

In all participants with diabetes (N = 2710), comparing with those without renal damage, subjects with reduced eGFR were older, more likely to be men, lower educated, unbefitting physical activity, insufficient consumption of fat and awareness of diabetes, higher proportion of hypertension and hyperuricemia, while those with albuminuria performed diet rich in fruits and vegetables, lacking control of diabetes and higher proportion of dyslipidemia (Table 3). One hundred and twenty six subjects were classified as stage 3–5 CKD and 908 subjects had albuminuria (Table 4). The prevalence of reduced eGFR was 4.7% (95% CI = 3.9–5.4%) and that of albuminuria was 33.5% (95% CI = 31.7–35.3%). The overall prevalence of CKD in participants with diabetes was 35.5% (95% CI = 33.7–37.3%).

**Table 3:** General characteristics of participants with diabetes according to indicators of renal damage.

	Participants with eGFR < 60 mL/min/1.73 m^2^ (N = 126)	Participants with albuminuria (N = 908)	Participants without renal damage (N = 1748)	Total (N = 2710)
Age	67.5 (12.6)	60.6 (10.7)	60.3 (10.0)	60.6 (10.4)
Men	59 (44.4%)	345 (38.0%)	634 (36.3%)	1003 (37%)
Education				
≤Primary school	85 (67.5%)	562 (61.9%)	1005 (57.5%)	1604 (59.2%)
Junior high school	21 (16.7%)	242 (26.7%)	505 (28.9%)	755 (27.9%)
≥Senior high school	20 (15.9%)	104 (11.5%)	237 (13.6%)	350 (12.9%)
^*^Per capita monthly income (RMB)				
≤500	35 (27.8%)	278 (30.6%)	591 (33.8%)	885 (32.7%)
500-	12 (9.5%)	226 (24.9%)	427 (24.4%)	656 (24.2%)
≥1000	37 (29.4%)	268 (29.5%)	550 (31.5%)	835 (30.8%)
Current smoker	18 (14.3%)	120 (13.2%)	219 (12.5%)	346 (12.8%)
Habitual drinker	12 (9.5%)	110 (12.1%)	215 (12.3%)	330 (12.2%)
Dietary pattern				
Diet rich in fruits and vegetables	44 (34.9%)	351 (38.7%)	625 (35.8%)	993 (36.7%)
High fat diet	8 (6.3%)	128 (14.1%)	269 (15.4%)	400 (14.8%)
Physical activity				
Low	81 (64.3%)	431 (47.5%)	718 (41.1%)	1185 (43.7%)
Moderate	15 (11.9%)	284 (31.3%)	635 (36.3%)	924 (34.1%)
High	30 (23.8%)	193 (21.3%)	394 (22.6%)	600 (22.1%)
Awareness of diabetes	62 (49.2%)	489 (53.9%)	1060 (60.7%)	1579 (58.3%)
Control of diabetes	57 (45.2%)	187 (20.6%)	531 (30.4%)	748 (27.6%)
Self-reported HBV infection	0 (0.0%)	5 (0.6%)	14 (0.8%)	19 (0.7%)
Hypertension	67 (53.2%)	493 (54.3%)	642 (36.7%)	1163 (42.9%)
Dyslipidemia	28 (22.2%)	446 (49.1%)	787 (45.0%)	1243 (45.9%)
Body mass index (kg/m^2^)	24.4 (3.7)	25.8 (3.8)	25.4 (3.6)	25.5 (3.7)
Total cholesterol (mmol/L)	5.1 (1.3)	5.1 (1.2)	4.9 (1.1)	4.9 (1.1)
Triglyceride (mmol/L)	2.0 (1.3)	2.4 (1.9)	2.1 (1.5)	2.2 (1.6)
LDL cholesterol (mmol/L)	3.1 (1.0)	3.0 (1.0)	3.0 (0.9)	3.0 (0.9)
HDL cholesterol (mmol/L)	1.4 (0.4)	1.2 (0.3)	1.3 (0.3)	1.3 (0.3)
FPG (mmol/L)	8.2 (3.5)	9.2 (3.1)	8.4 (3.0)	8.7 (3.1)
Uric acid (µmol/L)	342.4 (161.2)	280.3 (113.1)	266.6 (81.7)	272.4 (96.2)
Creatinine (µmol/L)	118 (100.8–166)	69.3 (40.6)	59.5 (14.0)	59 (50–71)
eGFR (mL/min/1.73 m^2^)	42.0 (15.4)	91.8 (20.9)	97.7 (12.8)	94.7 (17.8)
ACR (mg/g)	39.4 (17.0–176.3)	64.2 (41.3–122.2)	12.9 (7.8)	18.7 (9.2–41.7)

Note: Data were n (%), mean (standard deviation) or median with interquartile range, as appropriate.

Abbreviations: DKD, diabetic kidney disease; HBV, hepatitis B virus; LDL, low density lipoprotein; HDL, high density lipoprotein; eGFR, estimated glomerular filtration rate; FPG, fasting plasma glucose; ACR, albumin: creatinine ratio.

**Table 4:** Prevalence of indicators of renal damage in participants with diabetes, by disease stage.

Renal indicator						Diabetic kidney disease (in 2710 participants)	
Stage		eGFR (mL/min/1.73 m^2^)		Albuminuria			
		n	Prevalence (95% CI)	n	Prevalence (95% CI)	N	Prevalence (95% CI)
1	>90	1941	71.7 (70.0–73.3)	592	21.9 (20.3–23.4)	592	21.9% (20.3–23.4%)
2	60–89	643	23.7 (22.1–25.3)	244	9.0 (7.9–10.1)	244	9.0% (7.9–10.1%)
3	30–59	94	3.5 (2.8–4.2)	50	1.8 (1.3–2.4)	94	3.5% (2.8–4.2%)
3a	45–59	67	2.5 (1.9–3.1)	34	1.3 (0.8–1.7)	67	2.5% (1.9–3.1%)
3b	30–44	27	1.0 (0.6–1.4)	16	0.6 (0.3–0.9)	27	1.0% (0.6–1.4%)
4	15–29	21	0.8 (0.4–1.1)	17	0.6 (0.3–0.9)	21	0.8% (0.4–1.1%)
5	<15	11	0.4 (0.2–0.6)	5	0.2 (0.1–0.3)	11	0.4% (0.2–0.6%)
Total		2710	100	908	33.5 (31.7–35.3)	962	35.5% (33.7–37.3%)

Note: Albuminuria was defined as urinary albumin to creatinine ratio > 30 mg/g creatinine. Diabetic kidney disease was defined as a combination of diabetes and an eGFR less than 60 mL/min/1.73 m^2^ or presence of albuminuria.

Abbreviations: eGFR, estimated glomerular filtration rate.

Comparing with subjects with upper and lower tertiles of education and personal income, those with middle levels of education and personal income performed lower prevalences of eGFR less than 60 mL/min/1.73 m^2^ and albuminuria (Table 5). The adjusted prevalences of lower eGFR in subjects with junior high school education and 500–monthly personal income were 0.6% (95% CI = 0.5–0.8) and 0.5% (95% CI = 0.3–0.7%), and that of albuminuria were 12.9% (95% CI = 12.2–13.6%) and 13.5% (95% CI = 12.7–14.4%), respectively. Hence, the overall prevalence of CKD performed lowest in subjects with junior high school education (13.4%, 95% CI = 12.7–14.1%) and highest in subjects with primary school or lower education (21.0%, 95% CI = 10.1–21.9%). This trend similarly performed in subjects with different levels of personal income (17.0%, 95% CI 16.0–18.1% versus 13.8%, 12.9–14.7% versus 16.8%, 16.0–17.6%, *P*_trend_ < 0.001). The prevalences of hypertension, diabetes and DKD were positively associated with education and economic condition. All of them performed lowest in subjects with the upper tertile of education and personal income. The adjusted prevalences of DKD were 4.5% (95% CI = 4.0–5.0%), 2.3% (95% CI = 2.0–2.6%) and 1.5% (95% CI = 1.2–1.7%) in tertile 1–3 education (*P*_trend_ < 0.001), and 3.1% (95% CI = 2.6–3.6%), 2.6% (95% CI = 2.2–3.0) and 2.5% (95% CI = 2.1–2.8%), in tertile 1–3 economic condition (*P*_trend_ = 0.03), respectively.

**Table 5:** Adjusted prevalence of indicators of renal damage by education and economic development.

	eGFR < 60 mL/min/1.73 m^2^	Albuminuria	Hypertension	Diabetes	Awareness of diabetes	Control of diabetes	DKD	CKD
Education								
Primary school or lower, tertile 1	3.1 (3.5–4.2)	19.0 (18.1–19.9)	30.8 (29.7–31.8)	12.5 (11.8–13.3)	58.6 (56.2–61.0)	29.2 (27.0–31.4)	4.5 (4.0–5.0)	21.0 (20.1–21.9)
Junior high school, tertile 2	0.6 (0.5–0.8)	12.9 (12.2–13.6)	19.7 (18.9–20.6)	6.8 (6.2–7.3)	59.1 (55.6–62.6)	23.4 (20.4–26.5)	2.3 (2.0–2.6)	13.4 (12.7–14.1)
Senior high school or higher, tertile 3	2.4 (1.6–2.4)	13.4 (12.7–14.2)	7.6 (7.0–8.2)	4.8 (4.3–5.2)	55.1 (49.9–60.4)	29.4 (24.6–34.2)	1.5 (1.2–1.7)	15.1 (14.3–15.9)
*P_trend_	0.01	<0.001	<0.001	<0.001	0.38	<0.01	<0.001	<0.001
Per capita monthly income (RMB)								
≤500, tertile 1	2.0 (1.6–2.4)	15.7 (14.7–16.8)	27.0 (25.8–28.3)	11.8 (10.8–12.8)	62.4 (59.2–65.6)	25.1 (22.2–27.9)	3.1 (2.6–3.6)	17.0 (16.0–18.1)
500-, tertile 2	0.5 (0.3–0.7)	13.5 (12.7–14.4)	21.7 (20.6–22.8)	7.4 (6.7–8.1)	57.6 (53.8–61.4)	20.1 (17.0–23.2)	2.6 (2.2–3.0)	13.8 (12.9–14.7)
≥1000, tertile 3	1.9 (1.6–2.2)	15.7 (15.0–16.5)	17.0 (16.2–17.8)	7.5 (7.0–8.1)	54.1 (50.7–57.5)	25.7 (22.8–28.7)	2.5 (2.1–2.8)	16.8 (16.0–17.6)
*P_trend_	<0.001	<0.01	<0.001	<0.001	0.001	<0.01	0.03	<0.001

Note: Data were adjusted prevalence (%; 95% CI). Per capita monthly income was based on self-reported data.

Abbreviations: eGFR, estimated glomerular filtration rate; DKD, diabetic kidney disease; CKD, chronic kidney disease.

*P_trend_ was calculated by Mantel-Haenszel *chi-square* test.

Results of adjusted logistic regression demonstrated that increased age, gender (men versus women), habitual alcohol consumption, diabetes, hypertension and hyperuricemia were associated with higher risk of eGFR less than 60 mL/min/1.73 m^2^ with an OR of 1.98 (95% CI = 1.75–2.23), 1.32 (95% CI = 1.05–1.65), 1.78 (95% CI = 1.35–2.36), 2.30 (95% CI = 1.61–3.30), 1.40 (95% CI = 1.08–1.82), 1.30 (95% CI = 1.05–1.60) and 5.45 (95% CI = 4.29–6.93), respectively. Risk factors associated with albuminuria were diet rich in fruits and vegetables, unhealthy BMIs, diabetes, hypertension, dyslipidemia and hyperuricemia with an OR of 1.12 (95% CI = 1.04–1.22), 1.49 (95% CI = 1.21–1.84, underweight), 1.13 (95% CI = 1.04–1.23, overweight), 1.49 (95% CI = 1.29–1.72, obesity), 2.39 (95% CI = 2.16–2.64), 2.26 (95% CI = 2.08–2.45), 1.29 (95% CI = 1.19–1.40) and 1.43 (95% CI = 1.26–1.64), respectively. The lower tertile of education and upper tertile personal income were associated with higher risk of reduced eGFR and albuminuria (Table 6). Meanwhile, older age, lower education, overweight and obesity, hypertension and dyslipidemia were significantly associated with increasing risk of DKD with an OR of 1.23 (95% CI = 1.13–1.33), 1.20 (95% CI = 1.00–1.44), 1.44 (95% CI = 1.22–1.70), 1.87 (95% CI = 1.46–2.40), 2.20 (95% CI = 1.88–2.57), 2.51 (95% CI = 2.15–2.92), respectively (Table 6).

**Table 6:** Risk factors associated with indicators of renal damage and diabetic kidney disease

Age	eGFR < 60 ml/min/1.73 m^2^		Albuminuria		Diabetic kidney disease	
	Crude OR	Adjusted OR	Crude OR	Adjusted OR	Crude OR	Adjusted OR
18-	1.00	1.00	1.00	1.00	1.00	1.00
Age changed by 10 years	2.06 (1.89–2.24)	1.98 (1.75–2.23)	1.17 (1.14–1.21)	1.01 (0.97–1.05)	1.34 (1.26–1.42)	1.23 (1.13–1.33)
Gender						
Women	1.00	1.00	1.00	1.00	1.00	1.00
Men	1.05 (0.89–1.23)	1.32 (1.05–1.65)	0.90 (0.84–0.96)	1.00 (0.91–1.10)	0.91 (0.79–1.04)	1.00 (0.83–1.21)
Education						
Junior high school	1.00	1.00	1.00	1.00	1.00	1.00
≤Primary school	4.16 (3.26–5.30)	1.78 (1.35–2.36)	1.48 (1.37–1.60)	1.24 (1.13–1.36)	1.61 (1.38–1.87)	1.20 (1.00–1.44)
≥Senior high school	2.60 (1.93–3.51)	2.18 (1.47–3.24)	1.06 (0.95–1.18)	1.13 (0.99–1.29)	0.84 (0.66–1.05)	0.96 (0.73–1.27)
Per capita monthly income (RMB)						
500–1000	1.00	1.00	1.00	1.00	1.00	1.00
≤500	2.89 (2.16–3.87)	1.75 (1.29–2.36)	1.17 (1.07–1.29)	1.04 (0.94–1.15)	1.11 (0.93v1.33)	0.93 (0.77–1.12)
≥1000	2.42 (1.81–3.24)	2.50 (1.84–3.39)	1.11 (1.01–1.22)	1.11 (1.00–1.23)	0.95 (0.79–1.13)	0.97 (0.80–1.17)
Alcohol consumption	1.86 (1.46–2.38)	2.30 (1.61–3.30)	1.19 (1.09–1.30)	1.08 (0.96–1.22)	1.06 (0.90–1.26)	0.93 (0.74–1.16)
Diet rich in fruits and vegetables	0.73 (0.62–0.86)	1.09 (0.88–1.35)	0.94 (0.87–1.00)	1.12 (1.04–1.22)	0.83 (0.73–0.95)	1.08 (0.92–1.26)
High fat diet	0.34 (0.25–0.46)	0.46 (0.30–0.69)	0.74 (0.68–0.82)	0.85 (0.77–0.95)	0.74 (0.62–0.90)	0.92 (0.74–1.13)
Physical activity						
Low	1.00	1.00	1.00	1.00	1.00	1.00
Moderate	0.20 (0.16–0.26)	0.35 (0.27–0.46)	0.75 (0.69–0.81)	0.83 (0.76–0.91)	0.68 (0.59–0.80)	0.78 (0.66–0.93)
High	0.45 (0.37–0.55)	0.63 (0.50–0.81)	0.78 (0.72–0.85)	0.89 (0.80–0.98)	0.66 (0.56–0.79)	0.75 (0.62–0.91)
Body massv index						
Healthy weight	1.00	1.00	1.00	1.00	1.00	1.00
Underweight	1.85 (1.33–2.59)	1.16 (0.73–1.82)	1.38 (1.15–1.66)	1.49 (1.21–1.84)	0.98 (0.63–1.51)	0.77 (0.44–1.35)
Overweight	0.79 (0.66–0.95)	0.72 (0.57–0.90)	1.37 (1.28–1.48)	1.13 (1.04–1.23)	1.79 (1.54–2.07)	1.44 (1.22–1.70)
Obesity	1.08 (0.79–1.48)	0.93 (0.63–1.37)	2.14 (1.89–2.43)	1.49 (1.29–1.72)	2.60 (2.08–3.25)	1.87 (1.46–2.40)
Diabetes	1.98 (1.62–2.41)	1.40 (1.08–1.82)	2.99 (2.74–3.27)	2.39 (2.16–2.64)	NA	NA
Hypertension	2.02 (1.72–2.36)	1.30 (1.05–1.60)	2.62 (2.44–2.81)	2.26 (2.08–2.45)	3.05 (2.67–3.48)	2.20 (1.88–2.57)
Dyslipidemia	1.43 (1.22–1.68)	1.18 (0.95–1.46)	1.62 (1.51–1.73)	1.29 (1.19–1.40)	2.89 (2.52–3.30)	2.51 (2.15–2.92)
Hyperuricemia	5.10 (4.26–6.11)	5.45 (4.29–6.93)	1.79 (1.60–2.00)	1.43 (1.26–1.64)	1.59 (1.29–1.96)	0.96 (0.75–1.24)

Note: Data were crude and multivariable-adjusted odds ratio (95% Confidence Interval).

Abbreviation: DKD, diabetic kidney disease; NA, not applicable

Reference level: Gender = Woman; Education = Junior high school; Per capita monthly income = 500–1000; Alcohol consumption = No; Diet rich in fruits and vegetables = No; High fat diet = No; Physical activity = Low; Body mass index = Healthy weight; Diabetes = No; Hypertension = No; Dyslipidemia = No; Hyperuricemia = No.

In participants with diabetes, after adjusting for multiple confounders, age (OR = 1.61, 1.12–2.16), gender (men versus women, OR = 1.97, 1.15–3.37), top tertile of personal income (OR = 1.72, 1.01–2.95) and hyperuricemia (OR = 5.08, 2.82–9.17) were associated with increased risk of reduced eGFR. Factors associated with presence of albuminuria included gender (men, OR = 1.40, 1.13–1.74), diet rich in fruits and vegetables (OR = 1.25, 1.04–1.51), hypertension (OR = 1.99, 1.64–2.40) and dyslipidemia (OR = 1.20, 1.00–1.45). Moderate physical activity performed statistically preventive effects on reduced eGFR and albuminuria, with an OR of 0.23 (95% CI = 0.13–0.39) and 0.78 (95% CI = 0.65–0.93), respectively (Table 7). The results of collinearity diagnostics for factors in logistic regression showed that there was no collinearity between factors (Supplementary Tables 1 and 2).

**Table 7:** Risk factors associated with indicators of renal damage in participants with diabetes.

Age	eGFR < 60 ml/min/1.73 m^2^		Albuminuria	
	Crude OR	Adjusted OR	Crude OR	Adjusted OR
18-	1.00	1.00	1.00	1.00
Age changed by 10 years	1.72 (1.40–2.12)	1.61 (1.12–2.16)	1.02 (0.94–1.10)	0.94 (0.84–1.04)
Gender				
Women	1.00	1.00	1.00	1.00
Men	1.38 (0.96–1.98)	1.97 (1.15–3.37)	1.06 (0.90–1.26)	1.40 (1.13–1.74)
Education				
≤Primary school	1.00	1.00	1.00	1.00
Junior high school	0.51 (0.32–0.83)	0.88 (0.49–1.60)	0.88 (0.73–1.05)	0.89 (0.72–1.11)
≥Senior high school	1.08 (0.66–1.79)	0.47 (0.15–1.44)	0.78 (0.61–1.01)	0.77 (0.55–1.06)
Per capita monthly income (RMB)				
≤500	1.00	1.00	1.00	1.00
500–1000	0.45 (0.23–0.88)	0.69 (0.34–1.40)	1.15 (0.93–1.42)	1.21 (0.96–1.52)
≥1000	1.13 (0.70–1.81)	1.72 (1.01–2.95)	1.03 (0.84–1.26)	1.06 (0.85–1.32)
Alcohol consumption	1.33 (0.73–2.45)	1.75 (0.66–4.66)	0.99 (0.78–1.27)	1.13 (0.83–1.54)
Diet rich in fruits and vegetables	0.92 (0.64–1.34)	0.94 (0.56–1.58)	1.14 (0.97–1.34)	1.25 (1.04–1.51)
High fat diet	0.38 (0.18–0.78)	0.50 (0.19–1.30)	0.92 (0.74–1.16)	0.93 (0.72–1.20)
Physical activity				
Low	1.00	1.00	1.00	1.00
Moderate	0.23 (0.13–0.39)	0.52 (0.27–0.99)	0.78 (0.65–0.93)	0.84 (0.68–1.04)
High	0.72 (0.47–1.10)	1.08 (0.62–1.89)	0.83 (0.67–1.02)	0.90 (0.71–1.14)
Body mass index				
Healthy weight	1.00	1.00	1.00	1.00
Underweight	0.73 (0.22–2.41)	NA	1.19 (0.70–2.04)	1.27 (0.64–1.53)
Overweight	0.55 (0.36–0.84)	0.66 (0.38–1.14)	1.09 (0.90–1.33)	0.98 (0.79–1.22)
Obesity	0.51 (0.31–0.86)	0.81 (0.43–1.55)	1.41 (1.14–1.76)	1.15 (0.89–1.47)
Hypertension	1.54 (1.08–2.21)	1.48 (0.88–2.47)	2.01 (1.71–2.36)	1.99 (1.64–2.40)
Dyslipidemia	0.32 (0.21–0.49)	0.36 (0.20–0.63)	1.21 (1.03–1.42)	1.20 (1.00–1.45)
Hyperuricemia	4.46 (2.95–6.76)	5.08 (2.82–9.17)	1.55 (1.19–2.03)	1.16 (0.84–1.59)

Note: Data were crude and multivariable-adjusted odds ratio (95% Confidence Interval).

Abbreviation: DKD, diabetic kidney disease; NA, not applicable.

Reference level: Gender = Woman; Education = Primary school; Per capita monthly income = 500 –; Alcohol consumption = No; Diet rich in fruits and vegetables = No; High fat diet = No; Physical activity = Low; Body mass index = Healthy weight; Diabetes = No; Hypertension = No; Dyslipidemia = No; Hyperuricemia = No.

## Discussion

Henan province is one of the biggest provinces in China and has the most representative rural population accounting for an estimated 8% of the entire country according to the China Population Census in 2009. To the best of our knowledge, the present study was firstly performed in a large representative sample of Chinese rural population and evaluated the current epidemiological features of both CKD and DKD. In this study, the prevalence of chronic kidney disease was 16.4% and that of diabetic kidney disease was 2.9%, corresponding for over 12 million rural adults in Henan province. Generally, increased age, gender, unhealthy BMIs, diabetes, hypertension, dyslipidemia and hyperuricemia were significantly associated with higher risk of CKD and DKD. These findings were similar to results of previous studies and could be helpful for determining specific prevention strategies for CKD and DKD in Chinese rural population.

In a national survey using a multistage stratified sampling method in 2012, the prevalence of CKD in Chinese rural residents was reported to be 11.3%, in which 1.6% participants with reduced eGFR and 10.1% participants with albuminuria^18^. Comparing with our current study, number of people having CKD was markedly increased in the past five years. Older age was reported to be independently associated with increased risk of reduced renal function, further supported by our present study^11,21,23–26^. Aging has become a highlighted social problem in China. According to the data from China Population Census in 2009, the proportion of residents aged over 50 and 60 years was 24.0% and 12.7% in Henan province, respectively. In this study, the mean age of all subjects was 56.4 years old, which was almost 7 years older than that of participants from the national survey in 2012^18^. The difference in age distribution partly contributed to the higher prevalence of CKD in this study participants.

Hypertension and diabetes are also major risk factors of CKD^6,27–29^. A rapid increase in prevalence of hypertension and diabetes in China had occurred in the past decades. An early Chinese national survey of hypertension demonstrated that the overall prevalence of hypertension was 13.6% in residents who were older than 15 years old^30^. Ten years later, data from the International Collaborative Study of Cardiovascular Disease in Asia indicated that 27.2% of Chinese adults who aged 35–74 years had hypertension^31^. Results from the China Hypertension Survey, a nationwide screening conducted from 2012 to 2015, suggested that there was still 23.2% of Chinese adult population over 18 years of age had hypertension^32^. Similarly, a national survey reported the prevalence of diabetes, defining as a combination of questionnaire and standard 75-g oral glucose tolerance test, was 4.6% in 1998^33^. After that, data from the Fourth National Health and Nutrition Examination Survey of China (NHANES) indicated that the prevalence of diabetes had increased to 6.4% in 2004^34^. Even the rate of growth slowed down in the past decade, results of 2016 Global Burden of Disease study suggested that diabetes still affected 6.6% of all-age Chinese population^8^. In our current study, the prevalences of hypertension and diabetes were much higher than results from previous studies: 28.9% versus 23.2% and 11.4% versus 6.6%, respectively. Results of logistic regression also showed that hypertension and diabetes were associated with higher risk of reduced eGFR with ORs of 1.30 and 1.40, and of albuminuria with ORs of 2.26 and 2.39, respectively. Therefore, the higher prevalence of CKD in Henan rural population could be caused by the higher prevalences of hypertension and diabetes.

Hyperuricemia induced renal injury by its crystal-independent mechanisms, such as the activation of renin-angiotensin system, endothelial dysfunction and induction of oxidative stress^35^. A previous community-based cohort study of American population indicated that every 1 mg/dL increase of serum uric acid from baseline was associated with a 7% higher risk for decreased renal function, defined as an eGFR less than 60 mL/min/1.73 m^2^ ^36^. Another prospective cohort study conducted in Chinese population demonstrated that the increased level of uric serum acid was associated with both declination of eGFR and new-onset albuminuria^37^. A meta-analysis, combining 13 studies with over 190000 subjects with normal renal function at the baseline, suggested that hyperuricemia was contributed twofold increased risk of new-onset chronic kidney disease^38^. According to results of present study, comparing with subjects without renal damage, the prevalence of hyperuricemia was much higher in subjects with reduced eGFR and albuminuria: 27.9% and 11.6% versus 6.5%, respectively. The result of logistic regression further supported hyperuricemia was robustly connected with higher risk of reduced eGFR with an odds ratio of 5.45 (95% CI = 4.29–6.93) after adjusted for relevant covariates.

Of other factors related to renal damage, dyslipidemia tends to progress along with the declination of renal function in patients with CKD, even at the early stages of renal dysfunction and it also contributes to the association between CKD and cardiovascular disease, which is the main cause of death in patients with CKD and ESRD^39^. According to result of present study, the prevalence of dyslipidemia was 27.8%, similar to the latest national survey which reported a prevalence of 26.3% in Chinese rural residents^40^. Generally, dyslipidemia performed as a risk factor for albuminuria and diabetic kidney disease, with adjusted ORs of 1.29 and 2.51, respectively. However, in participants with diabetes, dyslipidemia was statistically associated with lower risk of reduced eGFR. For this special phenomenon, there were two potential explanations. Firstly, in all participants with diabetes, the proportion of dyslipidemia in subjects with reduced eGFR was 22.2%, while that was 49.1% and 45.0% in subjects with albuminuria and non-renal damage, respectively. The different statistical distributions might be partly contributed to the result of logistic regression. Secondly, in present study, the high fat diet was statistically associated with the lower risk of development of reduced eGFR and this dietary pattern is a main contributor to dyslipidemia. Nevertheless, it was still a risk factor for albuminuria with an OR of 1.20 in subjects with diabetes and deserved highly attention since its notable prevalence.

Sedentary lifestyle enhances the risk of development of many unhealthy conditions, such as hypertension, type 2 diabetes mellitus, osteoporosis and depression^41^. Chronic kidney disease patients are at high risk of premature death because of cardiovascular disease, which is partly contributed by their sedentary behavior. Previous studies demonstrated that increased physical activities could slow down the progress of declination of estimated glomerular filtration rate in patients with stage 3–4 CKD and improve their physical function, which could potentially contribute to lower risk of cardiovascular disease^42–44^. According to the results of the National Health and Nutrition Examination Survey III, comparing with the participants whose physical activity was defined as inactive, the hazard ratios of mortality for physically active participants was 0.59 in the non-CKD subpopulation and 0.44 in the CKD subpopulation^45^. Present study also supported that increased physical activity was associated with the lower risk of developing renal damage. Results of logistic regression indicated the odds ratios of reduced eGFR, albuminuria and DKD for moderate physical activity were 0.35, 0.83 and 0.78, respectively. However, in 2012, Hallal *et al*. reported that 31.1% of adult global population were physically inactive^46^. In 2015, it was estimated that 41% of the adults were defined as low total physical activity in France^47^. In present study, there were almost 40% of subjects performing insufficient physical activities. Hence, a public education program aiming at providing information about the benefits of moderate physical exercises and the recommendations of physical activity in general population should be promoted.

The present study reported the epidemiologic characteristics and influencing factors of chronic kidney disease and diabetic kidney disease based on a representative rural population in China. The standardized survey tools, training programs and the quality-control procedures ensured the reliability of the results. Nevertheless, several limitations should be addressed. First, the renal indicators of CKD and DKD were acquired from single measurements. The estimated prevalences of CKD and DKD might be overestimated. Second, the recognition of diabetes and hypertension was partly based on the usage of medicine and self-reported history. It might be affected by insufficient awareness rate. Finally, the cross-sectional study is incapable of evaluating causal relationships between indicators of renal damage and relevant influencing factors.

Results of present study demonstrate that chronic kidney disease and diabetic kidney disease revealed to a major burden of public health in Chinese rural population. The rapid increase prevalences of hypertension and diabetes will persistently affect the overall prevalence of chronic kidney disease in the future. It’s an urgent to develop specific strategies aiming at reduce the burden of CKD.

## Materials and Methods

### Study subjects

The subjects were recruited in 4 rural districts of Henan province in China from July 2015 to December 2017: Xuchang, Zhumadian, Luoyang and Zhengzhou. A multistage, stratified cluster sampling method was employed to select participants aged over 18 years old in general population. In the first stage, based on the consideration of local economic and medical condition and the quantity of population, 8 candidate regions were selected from 4 different geographical areas (east, south, west, and north) in Henan province. In the second stage, 1–3 representative rural districts in each region were selected by local Center for Disease Control and Prevention. In the final stage, all permanent residents who satisfied the inclusion criteria and agreed to sign the informed consent were recruited in this study. Altogether, a total of 25393 subjects aged 18 years or older were selected from 4 rural districts and 23869 subjects completed the survey and examination, with a response rate of 94.0%. This study was approved by the Zhengzhou University Life Science Ethics Committee (Code: [2015] MEC (S128)) and the Ethics Committee of the First Affiliated Hospital of Zhengzhou University (No. KY-2018-LW-66). All participants gave written informed consent before data collection. The present study was performed in accordance with the Declaration of Helsinki.

### Measurements and definitions

Data were collected by face-to-face interview in examination centers at local health stations. All subjects completed a questionnaire recording their sociodemographic status (e.g., age, gender, education and personal income, etc.), personal and family health history (e.g., diabetes, hypertension, hepatitis, etc.), awareness and control of chronic non-communicable disease (e.g., diabetes, hypertension, dyslipidemia, etc.), lifestyle (e.g., smoking, alcohol consumption, physical activity, etc.) and dietary pattern with assistance of medical students, trained practitioners, doctors and nurses. Anthropometric measurements, such as height, weight and blood pressure (BP), were obtained. Height and weight were measured in light clothing without shoes and body mass index (BMI, kg/m^2^) was calculated subsequently. According to the Chinese “Criteria of weight for adults (No. WS/T 428-2013, available on http://www.nhfpc.gov.cn)”, BMI was divided into four levels: underweight (<18.5), healthy weight (18.5–23.9), overweight (24–27.9) and obesity (≥28). Blood pressure was measured using electronic sphygmomanometer (Omron HEM-7071A, Japan) three times at one minute intervals. The mean value of three BP readings was used for statistical analysis, unless the difference between the readings was higher than 10 mm Hg, in which case the mean value of other two closest results was employed. Besides participants’ self-reported use of anti-hypertension medications in the past 2 weeks, hypertension was defined as participants whose average systolic blood pressure (SBP) ≥ 140 mm Hg, and/or average diastolic blood pressure (DBP) ≥ 90 mm Hg^48^.

After at least 8 hours of overnight fasting, venous blood specimen was collected in vacuum tubes without anticoagulation. Serum concentration of creatinine, uric acid, total cholesterol, triglyceride, high-density lipoprotein and low-density lipoprotein were measured using enzymatic colorimetry on Cobas C 701 (Roche). Fasting plasma glucose (FPG) level was estimated by glucose oxidative method (GOD-PAP). Urinary albumin and creatinine were measure from a fresh morning spot urine. Albuminuria was measured with immune-turbidimetric test. Urinary creatinine was evaluated by Jaffe’s kinetic method. The urinary albumin to creatinine ratio (ACR, mg/g) was calculated automatically.

Estimated glomerular filtration rate (eGFR) was calculated by the 2009 CKD-EPI creatinine equation^49^. Albuminuria was defined as participants with an ACR > 30 mg/g. Indicators of renal damage were presence of an eGFR less than 60 mL/min/1.73 m^2^ or albuminuria. Chronic kidney disease defined as participants with presence of one or two indicators of renal damage. Diabetes was defined according to the American Diabetes Association (ADA) 2009: 1. FPG ≥ 7.0 mmol/L; 2. Self-reported use of insulin or anti-diabetic medications in the past 2 weeks; 3. Self-reported a previous diagnosis of diabetes by physicians. Control of diabetes was defined as participants with diabetes kept their FPG less than 7.0 mmol/L. Diabetic subjects with albuminuria and/or eGFR less than 60 mL/min/1.73 m^2^ were classified as diabetic kidney disease. Dyslipidemia was presence of abnormal serum lipid concentrations, according to the Chinese guidelines on prevention and treatment of dyslipidemia in adults, or use of anti-dyslipidemia medications during last 2 weeks^50^. Hyperuricemia was defined as plasma uric acid concentration >422 µmol/L for man and >363 µmol/L for woman.

Awareness of those with hypertension and diabetes was considered if subjects reported having a previous diagnosis by physicians. Education were divided into 3 levels: 1. Primary school or lower; 2. Junior middle school; 3. Senior high school or above. Economic condition were also classified into 3 levels according to per capita monthly income (RMB): 1. ≤ 500 Yuan; 2. 500–999 Yuan; 3. ≥ 1000 Yuan. Data of personal income were not applicable in 3007 participants since they couldn’t properly evaluated their economic condition. Diet rich in fruits and vegetables was considered as daily average consumption more than 500 g. High fat diet was defined as daily average meat consumption of livestock and poultry more than 75 g^51^. According to the international physical activity questionnaire (IPAQ 2001), physical activity was classified as low, moderate and high degree^52^.

### Statistical analysis

Epidata software (version 3.1) was used for data entry and management. All statistical analyses were performed with SAS 9.1 (SAS Institute, Cary, NC, USA) and GraphPad Prism 6 (GraphPad Software, Inc., La Jolla, CA, USA) for Windows. A p-value < 0.05 was considered statistically significant. Data was expressed as mean ± SD., median with rang or frequency with percentage, as appropriate. Intergroup comparisons were made using Pearson *Chi-square* test for categorical variables and Student’s t-test, Mann-Whitney U-test or Wilcoxon test for continuous variables, as appropriate. Standard population of this study was using the data from the China Population Sampling Census in 2009 (data available on http://www.stats.gov.cn/).

The crude and adjusted prevalences of reduced eGFR (eGFR < 60 mL/min/1.73 m^2^), albuminuria, diabetic kidney disease and chronic kidney disease were reported. Staging of CKD based on the Kidney Disease Outcome Quality Initiative guideline and the stage 3 CKD was further divided into stage 3 A and stage 3B with an eGFR of 45 mL/min/1.73 m^2^ as the cut-off value^53^. We further stratified the prevalences of two renal damage indicators and DKD by economic and education condition. Prevalences of hypertension, diabetes, awareness and control of diabetes were reported.

Logistic regression was employed to explore the association between indicators of renal damage and relevant covariates. Both the crude and multivariable adjusted odds ratios (ORs) with 95% confidential intervals (CIs) were reported. Covariates involved in our multivariable logistic regression model were age (with 10 years interval), gender, education (≤primary school versus junior high school [reference] versus ≥ senior high school), economic condition (≤500 versus 500 - [reference] versus ≥ 1000), alcohol consumption (yes versus no), BMI (healthy weight [reference] versus underweight versus overweight versus obesity), diabetes (yes versus no), hypertension (yes versus no), dyslipidemia (yes versus no), hyperuricemia (yes versus no). Multicollinearity between covariates was calculated by using multi linear regression method.

## Supplementary information

Supplementary Materials

## Data Availability

The datasets generated during and/or analysed during the current study are available from the corresponding author on reasonable request.
