# Supplementary material for: Prevalence and risk factors of chronic kidney disease and diabetic kidney disease in Chinese rural residents: a cross-sectional survey
Source: Sci Rep. 2019 Jul 18;9:10408. doi: 10.1038/s41598-019-46857-7 (PMC6639314; doi:10.1038/s41598-019-46857-7)
Supplement: Supplementary file 1 — Supplementary Materials [file 41598_2019_46857_MOESM1_ESM.docx]

Prevalence and risk factors of chronic kidney disease and diabetic kidney disease in Chinese rural residents: a cross-sectional survey

Jiayu Duan^1^, Chongjian Wang^2^, Dongwei Liu^1^, Yingjin Qiao^1^, Shaokang Pan^1^, Dengke Jiang^1^, Zihao Zhao^1^, Lulu Liang^1^, Fei Tian^1^, Pei Yu^1^, Yu Zhang^1^, Huanhuan Zhao^1^, Zhangsuo Liu^1^*

Authors affiliations:

^1^ Department of Nephrology, The First Affiliated Hospital of Zhengzhou University, Research Institute of Nephrology, Zhengzhou University, Zhengzhou 450052, China

^2^ Department of Epidemiology and Biostatistics, College of Public Health, Zhengzhou University, Zhengzhou, Henan, PR China.

* Corresponding author

Prof. Zhangsuo Liu

Department of Nephrology

The First Affiliated Hospital of Zhengzhou University, Research Institute of Nephrology

Zhengzhou University

Jianshe Road No.1, Zhengzhou, 450052, Henan, PR China

Phone: +86-0371-66913063

Fax: +86-0371-66970906

E-mail: zhangsuoliu@zzu.edu.cn

| S. Table 1 Results of collinearity diagnostics for factors in logistic regression of CKD in all participants | | |
| --- | --- | --- |
| Factors | Tolerance | VIF |
| Age | 0.87 | 1.15 |
| Gender | 0.71 | 1.42 |
| Education | 0.97 | 1.03 |
| Per capita monthly income | 0.97 | 1.03 |
| Alcohol consumption | 0.70 | 1.42 |
| Diet rich in fruits and vegetables | 0.94 | 1.07 |
| High fat diet | 0.92 | 1.08 |
| Physical activity | 0.98 | 1.03 |
| Body mass index | 0.91 | 1.11 |
| Diabetes | 0.96 | 1.04 |
| Hypertension | 0.89 | 1.12 |
| Dyslipidemia | 0.92 | 1.08 |
| Hyperuricemia | 0.96 | 1.04 |
| Abbreviations: CKD, chronic kidney disease; VIF, variance inflation factor. | | |

| S. Table 2 Results of collinearity diagnostics for factors in logistic regression of CKD in participants with diabetes | | |
| --- | --- | --- |
| Factors | Tolerance | VIF |
| Age | 0.80 | 1.25 |
| Gender | 0.75 | 1.33 |
| Education | 0.75 | 1.33 |
| Per capita monthly income | 0.92 | 1.08 |
| Alcohol consumption | 0.77 | 1.29 |
| Diet rich in fruits and vegetables | 0.95 | 1.06 |
| High fat diet | 0.90 | 1.11 |
| Physical activity | 0.96 | 1.05 |
| Body mass index | 0.90 | 1.11 |
| Hypertension | 0.89 | 1.12 |
| Dyslipidemia | 0.94 | 1.06 |
| Hyperuricemia | 0.96 | 1.04 |
| Abbreviations: CKD, chronic kidney disease; VIF, variance inflation factor. | | |
